# Inflammation marker ESR is effective in predicting outcome of diffuse large B-cell lymphoma

**DOI:** 10.1186/s12885-018-4914-4

**Published:** 2018-10-19

**Authors:** Shuang Wu, Ye Zhou, Hai-Ying Hua, Yan Zhang, Wen-Yan Zhu, Zhi-Qing Wang, Jin Li, Hua-Qiang Gao, Xiao-Hong Wu, Ting-Xun Lu, Dong Hua

**Affiliations:** 1 Department of Hematology, The Third Affiliated Hospital of Nantong University, The Third People’s Hospital of Wuxi, Wuxi, 214000 Jiangsu People’s Republic of China; 2 Department of Oncology, Affiliated Hospital of Jiangnan University, Wuxi, 214000 Jiangsu People’s Republic of China

**Keywords:** Diffuse large B-cell lymphoma, Erythrocyte sedimentation rate, Prognosis, Survival

## Abstract

**Background:**

Systemic inflammation has been implicated in cancer development and progression. This study examined the best cutoff value of erythrocyte sedimentation rate (ESR) in diffuse large B-cell lymphoma (DLBCL) patients.

**Methods:**

The relationship between ESR and clinical characteristics was analyzed in 182 DLBCL patients from 2006 to 2017. The log-rank test, univariate analysis, and Cox regression analysis were applied to evaluate the relationship between ESR and survival. An ESR of more than 37.5 mm/hour was found to be the optimal threshold value for predicting prognosis.

**Results:**

ESR was associated with more frequent advanced Ann Arbor stage, poorer performance status, elevated lactate dehydrogenase level, the presence of B symptoms, high-risk International Prognostic Index (IPI 3–5), more extranodal involvement (ENI ≥2), non-germinal-center B-cell (non-GCB) subtypes, and more frequent Myc protein positivity. Shorter overall survival (OS) and progression-free survival (PFS) were found for patients with higher ESRs. Multivariate analysis demonstrated that ESR level is an independent prognostic factor of both OS and PFS. In addition, dynamic changes in ESR are valuable in assessing curative effect and predicting disease recurrence.

**Conclusion:**

High ESR in DLBCL patients indicated unfavorable prognosis that may require alternative treatment regimens.

**Electronic supplementary material:**

The online version of this article (10.1186/s12885-018-4914-4) contains supplementary material, which is available to authorized users.

## Background

Diffuse large B-cell lymphoma (DLBCL) is one of the most prevalent non-Hodgkin lymphomas (NHLs) which are heterogeneous both clinically and genetically. With current immunochemotherapy, approximately 30% of patients fail chemotherapy [1].

Despite recent progress in understanding the molecular biology of DLBCL, clinical risk factor models are still used to identify patients who are unlikely to be cured with current therapy. The most widely used model is the International Prognostic Index proposed by the National Comprehensive Cancer Network (NCCN-IPI), which is based on clinical parameters [2].

Although the NCCN-IPI is robust and confirmed to be reproducible [3–5], the link between the included clinical parameters and underlying biology or targeted treatment remains to be defined [6]. Therefore, molecular markers with great prognostic significance in DLBCL are being used, but it is costly and most rely on tissue. Therefore, alternative readily available prognostic characteristics with low clinical cost are greatly needed to improve risk assessment for individual patients.

There is thus a need for powerful, independent prognostic and predictive factors that can be analyzed using non-invasive methods such as with the patient’s serum that can enable individualized treatment modalities for patients with poor prognosis.

Cancer has been regarded as a wound that does not heal, and inflammation has long been recognized as being important, playing a critical role in various processes related to cancer progression [7]. Most tumors are highly infiltrated by immune cells, including macrophages, neutrophils and lymphocytes. Thus, inflammation and host immune response-related markers may be relevant as biological markers of DLBCL progression [8], although they have not been investigated to a great extent and not with respect to their relevance for the survival of patients with DLBCL.

In the past, a number of laboratory markers have been proposed for prognosis in DLBCL. Among these, systemic inflammation has been implicated in cancer development and progression [9, 10]. ESR is routinely measured in clinical practice for inpatients, as an indicator of infection, sepsis, or autoimmunity and malignancy as well [11–17]. Increased ESR values have, for example, been found to correlate with overall poor prognosis in Hodgkin’s disease, breast, glioma, gastric and colorectal, prostate and renal cell carcinoma, as well as in Mycosis fungoides [14, 16–18]. It was believed that elevated ESR indicates a greater risk of NHLs [8]. However, the prognostic value of elevated ESR in DLBCL patients has not been systematically explored. In this study, therefore, we analyzed the clinical significance and the prognostic value of elevated ESR in DLBCL patients.

## Methods

### Selection of patients

According to the 2016 World Health Organization (WHO) classification, we reviewed the medical records of patients who were diagnosed with de novo DLBCL at the third affiliated hospital of Nantong University and affiliated Hospital of Jiangnan University from 2006 to 2017. Only patients treated with R-CHOP (rituximab plus cyclophosphamide, doxorubicin, vincristine, and prednisone) or R-CHOP-like chemotherapy were included. Patients with primary central nervous system lymphoma, transformed NHL, post-transplant lymphoproliferative disorders, primary mediastinal B-cell lymphoma, and HIV-positive DLBCL were excluded from the study. In addition, patients with clinical evidence of anemia, chronic kidney disease, congestive heart failure, acute infection or chronic inflammatory disease were also excluded [12]. A total of 182 patients with DLBCL ultimately qualified for the study.

### Cutoff ESR value

The optimal ESR cutoff value was 37.5 mm/hour as determined by X-tile software (Additional files 1 and 2: Figures S1 and S2).

### Immunohistochemistry

Immunohistochemistry was performed on 4-μm FFPE sections. Antibodies used in the study were CD20 (clone L26, Abcam, cutoff: 30%), CD10 (clone 56C6, Dako, cutoff: 30%), MUM1 (clone MUM1p, Dako), Bcl6 (clone LN22, Dako, cutoff: 30%), Myc (clone Y69; Abcam, cutoff: 40%) and Bcl2 (clone 124; Dako, cutoff: 50%). Cutoff scores for each antibody were described previously [2, 19].

### Statistical analysis

Overall survival (OS) and progression free survival (PFS) were defined as in Cheson 2014 [20]. Survival curves were plotted using Kaplan-Meier method and were compared by log-rank test. Statistical analysis was performed using SPSS software, version 20.0. The Chi-squared and Fisher exact tests were used to determine differences in frequencies between groups. Cox regression model and multivariate analyses were performed in this study. For all the tests, a probability value of less than 0.05 (2-sided) was considered statistically significant.

## Results

### Patients’ characteristics

The median age of the whole cohort was 55 years (range: 22 to 85). The prevalence of elevated ESR was 33.0% (60/182). The baseline clinical parameters of patients are presented in Table 1.

**Table 1:** The clinical characteristics of the182 patients of DLBCL

Characteristics	No. of cases (%)
Age (years)	
≤ 60	114 (62.6)
Male	110 (60.4)
Stage III-IV	111 (61.0)
Elevated ESR	61 (33.5)
Elevated LDH	84 (46.2)
ECOG PS ≥ 2	30 (16.5)
ENI ≥ 2	45 (24.7)
IPI score of 3–5	49 (26.9)
B symptoms	85 (46.7)
COO (Hans)	
GCB	73 (40.1)

*Abbreviations*: *COO*: cell of origin; *DLBCL*: diffuse large B-cell lymphoma; *ECOG PS*: performance status of Eastern Cooperative Oncology Group; *ENI*: extranodal involvement; *ESR*: erythrocyte sedimentation rate; *GCB*: germinal-center B-cell type; *IPI:* International Prognostic Index; *LDH*: lactate dehydrogenase

### Association between clinical features and ESR

In the whole cohort, ESR positivity was significantly associated with more frequent advanced Ann Arbor stage (*p* = 0.0002), poorer performance status (PS) (*p* < 0.0001), elevated LDH level (*p* < 0.0001), presence of B symptoms (*p* < 0.0001), high-risk IPI (IPI 3–5) (*p* < 0.0001), more extranodal involvement (ENI ≥ 2) (*p* = 0.024), non-GCB subtypes (*p* = 0.0004) and more frequent Myc protein positivity (*p* = 0.006) (Table 2).

**Table 2:** Associations between clinical features and ESR

Characteristics	ESR^+^	ESR^−^	*P* value
	No. of cases (%)		
Age (years)			
≤ 60	37	77	0.849
> 60	23	45	
Sex			
Male	36	74	0.932
Female	24	48	
Stage			
III-IV	48	63	0.0002
I-II	12	59	
Myc			
Positive	28	32	0.006
Negative	32	90	
ECOG PS			
≥ 2	20	10	< 0.0001
< 2	40	112	
LDH			
Over ULN	47	37	< 0.0001
normal	13	85	
ENI			
≥ 2	21	24	0.024
< 2	39	98	
Bcl2			
≥ 70%	30	56	0.603
< 70%	30	66	
IPI			
3–5	31	19	< 0.0001
0–2	29	103	
B symptoms			
Positive	43	42	< 0.0001
Negative	17	80	
COO (Hans)			
GCB	13	60	0.0004
Non-GCB	47	62	
DEL			
Positive	14	19	0.202
Negative	46	103	

Abbreviations: COO: cell of origin; DEL: double expression lymphoma; ECOG PS: performance status of Eastern Cooperative Oncology Group; ENI: extranodal involvement; ESR: erythrocyte sedimentation rate; GCB: germinal-center B-cell type; IPI: International Prognostic Index; LDH: lactate dehydrogenase; ULN: upper limit of normal

### Survival analysis of ESR

**Fig. 1:**
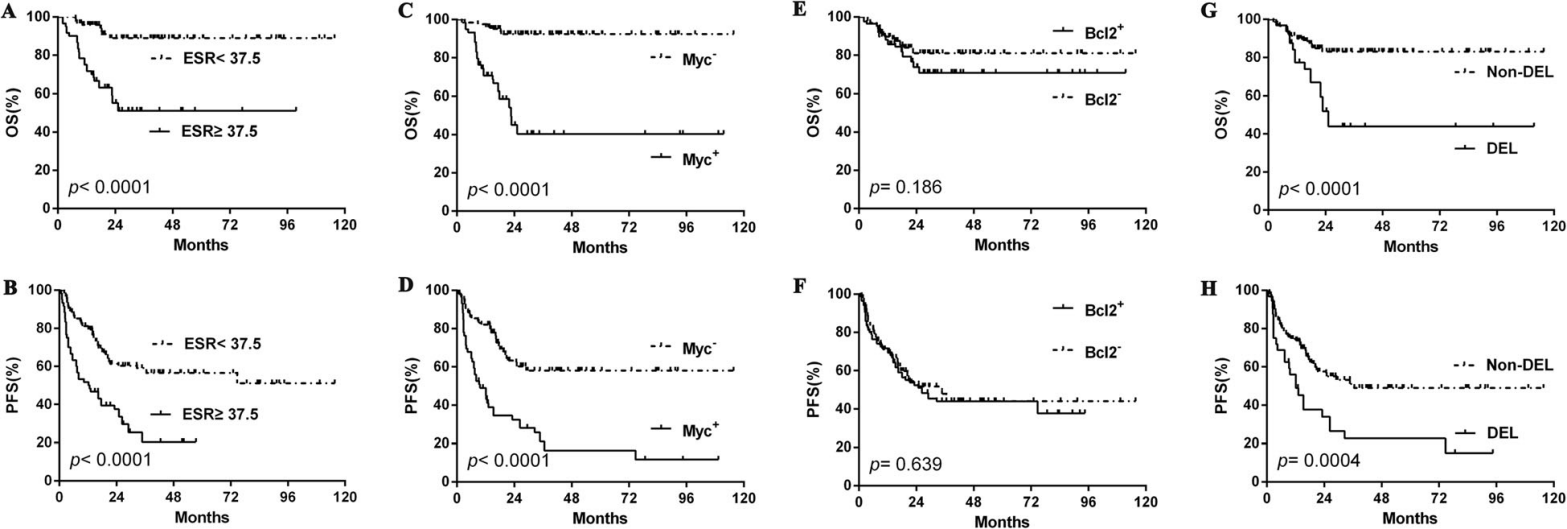
The differences of overall survival and progression-free survival in cases grouped according to ESR (**a**-**b**), Myc protein positivity (**c**-**d**), Bcl2 protein positivity (**e**-**f**), and DEL (**g**-**h**). Abbreviations: ESR: erythrocyte sedimentation rate; OS: overall survival; PFS: progression-free survival; DEL: double expression lymphoma

**Fig. 2:**
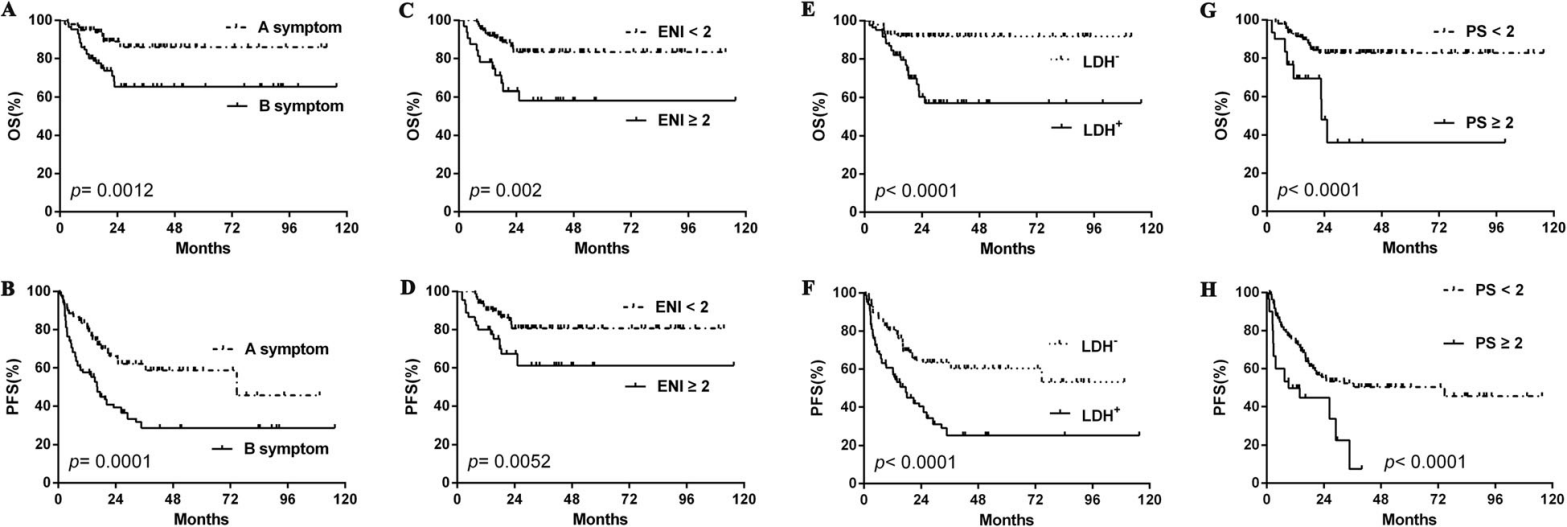
The differences of overall survival and progression-free survival in cases grouped according to B symptom (**a**-**b**), ENI (**c-d**), LDH level (**e**-**f**), PS status (**g**-**h**). Abbreviations: ENI: extranodal involvement; LDH: lactate dehydrogenase; PS: performance status of Eastern Cooperative Oncology Group; OS: overall survival; PFS: progression-free survival

**Fig. 3:**
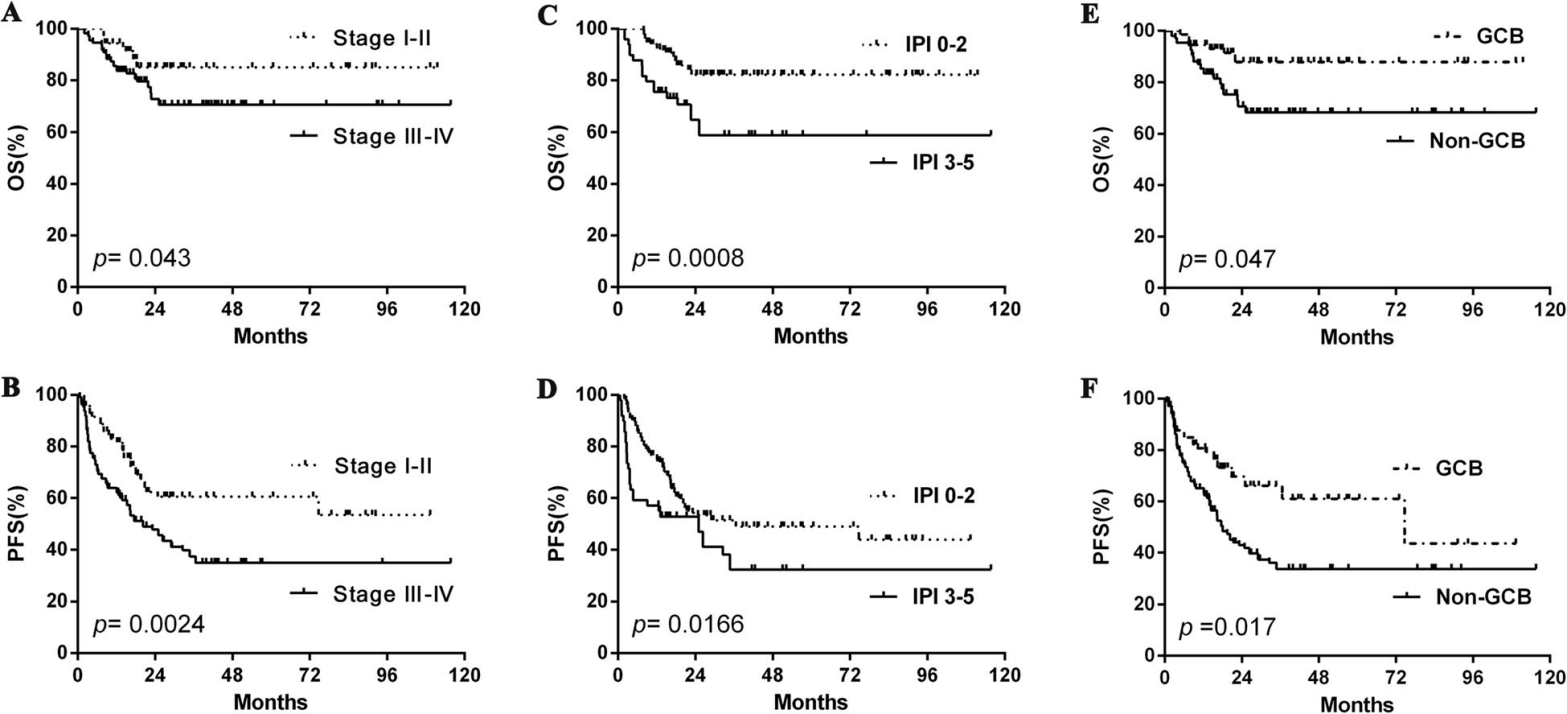
The differences of overall survival and progression-free survival in cases grouped according to clinical stage (**a-b**), IPI risk stratification (**c-d**) and COO (**e-f**). Abbreviations: IPI: International Prognostic Index; GCB: germinal-center B-cell type; OS: overall survival; PFS: progression-free survival

To expand our findings, survival analyses were performed on the cohort. Similar to what was found for clinical features, ESR-positive patients showed significantly decreased OS compared to ESR-negative ones (2-year OS rate, 55.2% vs. 89.0%, *p* < 0.001) and PFS (2-year PFS rate, 37.5% vs. 60.3%, *p* < 0.001) (Fig. 1a-1b). Other clinical and pathological factors were also relevant to lower survival ratio, including Myc protein positivity, double expression lymphoma (DEL), B symptoms, ENI ≥ 2, elevated LDH level, poor PS, advanced Ann Arbor stage, high-risk IPI, and non-GCB subtype (Fig. 1c-1h, Fig. 2a-2h, Fig. 3a-3f). Multivariate analysis, including ESR and the above clinical and pathological parameters, found that abnormal ESR is one of the independent prognostic factors for OS (HR: 1.897; 95% CI = 1.180–2.950 *p* = 0.037) and PFS (HR: 1.713; 95% CI = 1.090–2.261 *p* = 0.043) (Table 3).

**Table 3:** Univariate and multivariate analysis of clinical factors for OS and PFS

Variate	multivariate analysis (OS)				multivariate analysis (PFS)			
	HR	95% CI		*P* value	HR	95% CI		*P* value
Non-GCB	0.746	0.299	1.862	0.531	1.630	1.377	2.050	0.076
Myc positivity	1.629	1.057	3.291	< 0.001	1.650	1.227	2.540	< 0.001
B symptom	1.509	1.235	2.104	0.088	0.710	0.425	1.184	0.190
ECOG PS ≥ 2	0.708	0.568	1.015	0.528	0.937	0.521	1.687	0.829
ENI ≥ 2	0.494	0.195	1.251	0.137	1.623	1.236	2.760	0.004
Stage III-IV	1.057	0.534	1.447	0.521	0.768	0.451	1.309	0.333
Elevated LDH	1.891	1.221	2.178	0.094	1.774	1.437	2.371	0.079
IPI 3–5	2.070	1.088	2.550	0.046	2.071	1.066	3.025	0.032
ESR ≥ 37.5 mm/hour	1.897	1.180	2.950	0.037	1.713	1.090	2.261	0.043

*Abbreviations*: *ECOG PS*: performance status of Eastern Cooperative Oncology Group; *ENI:* extranodal involvement; *ESR*: erythrocyte sedimentation rate; *GCB*: germinal-center B-cell type; *LDH*: lactate dehydrogenase; *IPI*: International Prognostic Index; *OS*: overall survival; *PFS*: Progression-free survival

### Dynamic changes in ESR and clinical efficacy

**Fig. 4:**
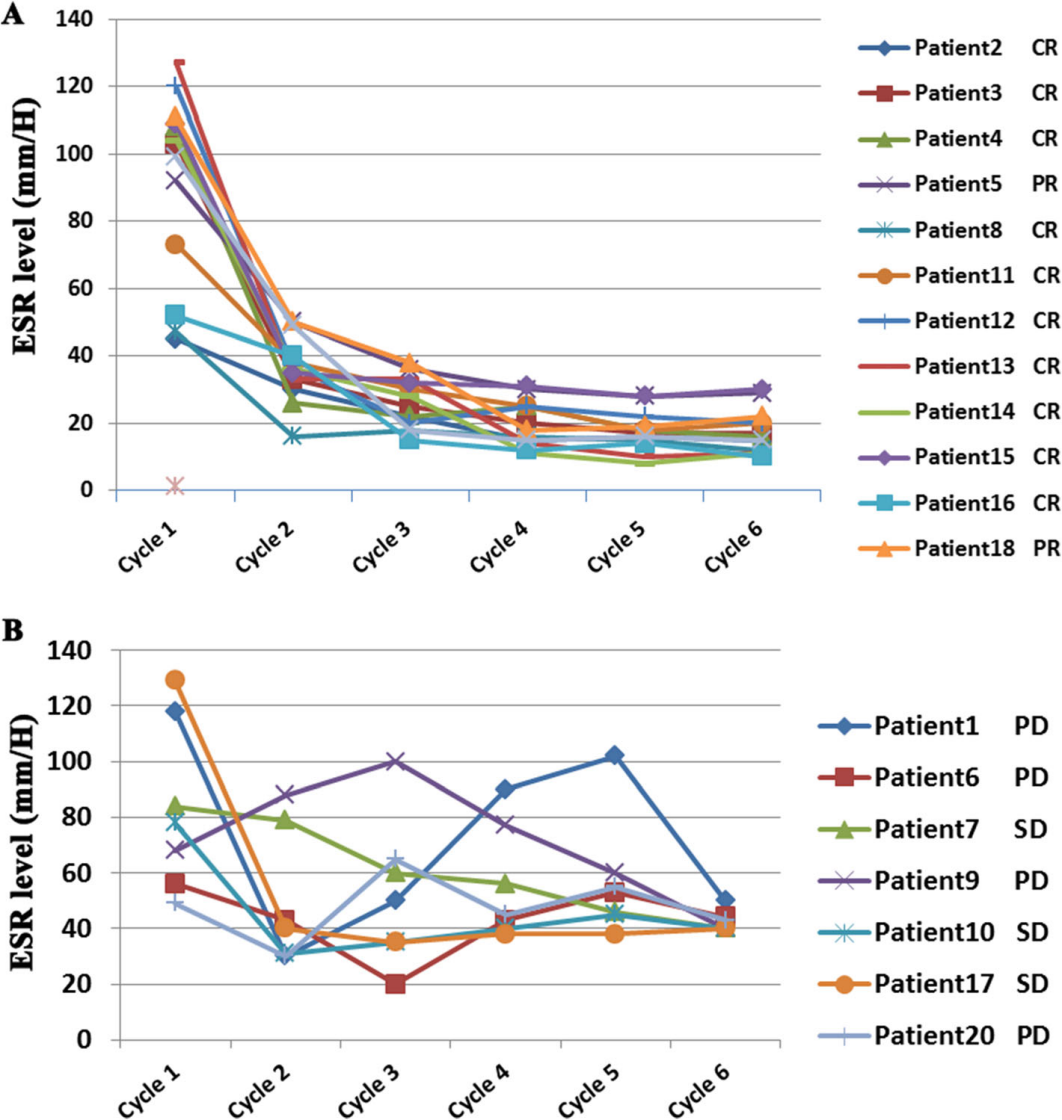
Dynamic changes in ESR and clinical efficacy. ESR in most patients (8/10) who achieved CR fell below cutoff value after the first cycle and had never risen above 37.5 mm/hour again. Similarly, in PR group, ESR value of all the patients (3/3) were dropped below cutoff value after two or three cycles. In contrast, ESR of patients in the stable disease/ progressive disease (SD/PD) group almost stayed above cutoff value or rebounded after the initial two cycles. Abbreviations: CR: Complete remission; PR: Partial response; SD: Stable disease; PD: Progressive disease

A total of 20 patients who completed 6 cycles of standard treatment were chosen for dynamic analysis. The association between ESR and clinical efficacy was analyzed in different treatment cycles. We noticed that ESR in most patients (8/10) who achieved complete remission (CR) fell below the cutoff level after the first cycle and had never risen above 37.5 mm/hour again (Fig. 4a). Similarly, in the partial response (PR) group, the ESR of all patients (3/3) had dropped below the cutoff level after two or three cycles (Fig. 4a). In contrast, ESR of patients in the stable disease/progressive disease (SD/PD) group almost stayed above the cutoff levels or rebounded after the initial two cycles (Fig. 4b).

## Discussion

In this study, we evaluated the prognostic value of ESR in DLBCL patients. Our results showed that ESR was a reliable factor predicting the outcome of DLBCL. ESR, obtained at diagnosis, is a novel and immediate prognostic factor in DLBCL patients. This is the first time that the prognostic value of serum ESR in DLBCL patients is discussed in the literature. ESR at diagnosis is significantly related high-risk clinical features in patients who received rituximab-based chemotherapy. In addition, ESR could be used as a monitoring biomarker. We provide evidence that a higher ESR is associated with poorer outcomes than lower ESR; these patients may require more aggressive treatment regimens.

Throughout the processes of most biological behaviors of cancer, inflammation plays an important role [21]. The first recognition of the relationship between inflammation and tumor growth was made in the nineteenth century, and is considered as one of the hallmarks of cancer [22]. An increasing amount of evidence indicates that the majority of tumors are linked to chronic inflammation [23]. Chronic inflammation can give rise to a mutagenic microenvironment which is either initiating cancer transformation or promoting gene mutation [21].

It is widely accepted that close relationships exist between infection and diseases, such as of Helicobacter pylori infection and gastric cancer or mucosa-associated lymphoid tissue lymphoma; Hepatitis B or C viruses and hepatocellular carcinoma; and Schistosoma or Bacteroides and bladder or colon cancer [24–26]. Additionally, many non-specific inflammatory markers also participated in cancer development. For example, C-reactive protein is a biomarker of acute inflammation, while ESR is a marker of chronic inflammatory conditions [27]. Both play key roles in cancer development and progression [24], and persistent of chronic infection plays a more important role in cancer progression [21]. Therefore, ESR, a chronic inflammatory marker, is more suitable for tracking inflammation among patients with chronic conditions [8]. Cumulative research has shown that high ESR is a significant predictor for cancer-specific survival of solid tumors [12, 14, 16].

Recently, inflammatory processes have been identified to play an important role in the pathogenesis of lymphoma [28, 29], and circulating inflammatory parameters were associated with a poor prognosis in DLBCL [8, 30, 31]. A retrospective study showed that modified Glasgow prognostic scores (mGPS) could be used as a predictor in DLBCL treated with R-CHOP regimens [27]. Patients with lower mGPS had higher CR rates and better OS. So far, few studies, including the above mGPS study, examined ESR, which is one of the most commonly used laboratory markers of chronic inflammation [27]. One preliminary case-control study evaluated the diagnostic value of ESR in differentiating active Crohn’s disease (ACD) from intestinal lymphoma and discovered ESR was lower in the ACD group, compared with the lymphoma group [32], which indicated that ESR has a more important function in lymphoma development than other inflammatory disease. Another study analyzed several inflammation markers primary gastric DLBCL patients, including ESR, and ultimately considered beta-2 microglobulin, but not ESR, was related to poor outcomes in DLBCL patients [8]. Unfortunately, it was a rather small study that only included 49 patients, and the significance remains to be confirmed. Therefore, the prognostic value of serum ESR in patients with malignant lymphoma, especially DLBCL, is still uncertain. Based on this, we enlarged the sample size, reanalyzed the prognostic value of ESR, and found that ESR is a powerful biomarker predicting poor prognosis for DLBCL patients.

As chronic inflammatory processes affect all stages of tumor development as well as therapy [21], only prognostic significance is just not enough. The dynamic change of chronic inflammatory marker on therapeutic effect evaluation and recurrence forecast seems to be more important. ESR has been confirmed to be helpful in monitoring chronic inflammatory conditions [27]. Elevated ESR is associated with increased mortality in patients with dermatomyositis due to respiratory failure [12]. Thus, monitoring ESR should be an integral part of the clinical care of dermatomyositis patients. In addition, dynamic change of the systemic immune inflammation index with hepatocellular carcinoma predicts prognosis after curative resection. In our study, we evaluated the dynamic change of ESR in DLBCL. We observed that ESR in most patients in the CR/PR group fell below the cutoff value after the initial cycles and was never above the cutoff value again, whereas ESR in patients in the SD/PD group almost stayed above cutoff levels or rebounded after the initial cycles. We determined that ESR prior to treatment is a promising factor for monitoring treatment response and disease status.

## Conclusions

This study is limited by its retrospective design and relatively small number of patients. Further prospective studies including more patients are required. In spite of these limitations, our study suggests that pretreatment ESR is associated with OS and PFS in DLBCL patients treated with immunochemotherapy. High ESR before treatment initiation was a reliable prognostic factor of unfavorable prognosis in DLBCL patients. Also, dynamic changes in ESR are valuable in assessing curative effect and predicting disease recurrence. Building on our findings, we recommend that ESR be used as an inexpensive biomarker for risk assessment in patients with DLBCL that can be determined without difficulty. Patients with high ESR after initial cycles might need more aggressive or second line therapies.

## Additional files

Additional file 1: **Figure S1.** The optimal cutoff value was 39 mm/hour for ESR with OS according to X-tile. Abbreviations: ESR: erythrocyte sedimentation rate; OS: overall survival. (TIF 158 kb)

Additional file 2: **Figure S2.** The optimal cutoff value was 36 mm/hour for ESR with PFS according to X-tile. Abbreviations: ESR: erythrocyte sedimentation rate; PFS: progression-free survival. (TIF 154 kb)

## Abbreviations

ACD: Active Crohn’s disease
CR: Complete remission
DEL: Double expression lymphoma
DLBCL: Diffuse large B cell lymphoma
ENI: Extranodal involvement
ESR: Erythrocyte sedimentation rate
HR: Hazard ratio
IPI: International prognostic index
LDH: Lactate dehydrogenase
mGPS: Modified Glasgow prognostic scores
NCCN-IPI: International prognostic index of national comprehensive cancer network
NHL: Non-Hodgkin lymphoma
OS: Overall survival
PD: Progressive disease
PFS: Progression-free survival
PR: Partial response
PS: Performance status
R-CHOP: Rituximab plus cyclophosphamide, doxorubicin, vincristine, and prednisone
SD: Stable disease
WHO: World Health Organization
