# Supplementary figures and images for: Inflammation marker ESR is effective in predicting outcome of diffuse large B-cell lymphoma
Source: BMC Cancer. 2018 Oct 19;18:997. doi: 10.1186/s12885-018-4914-4 (PMC6194702; doi:10.1186/s12885-018-4914-4)

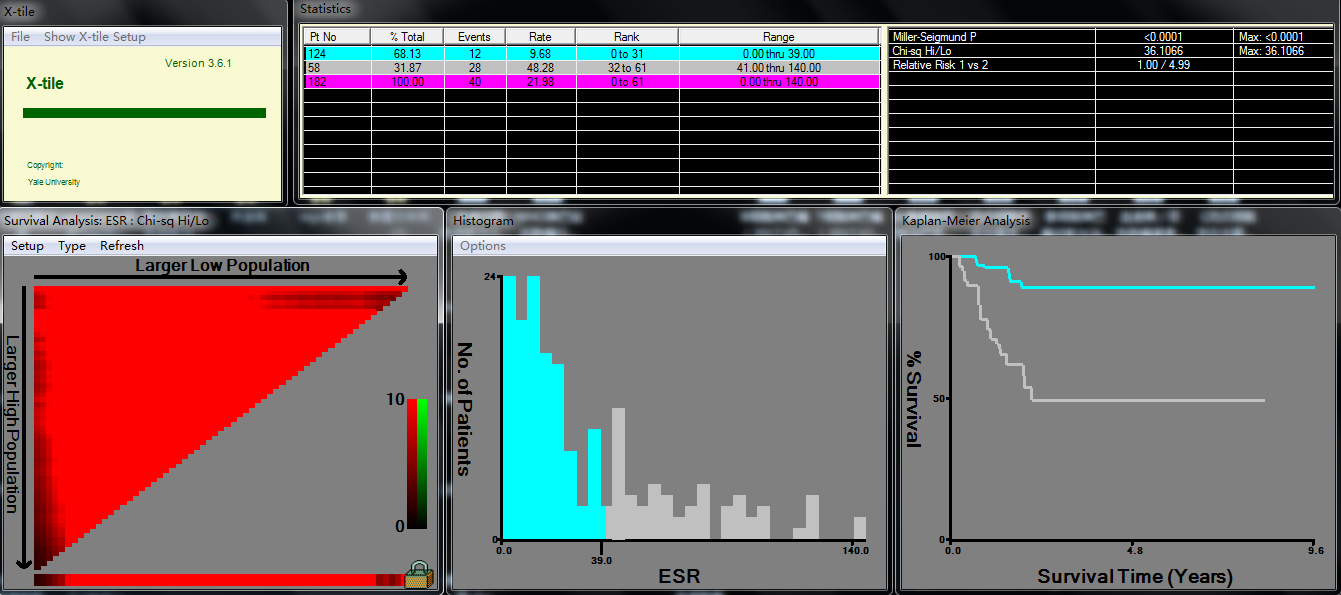

Supplement: Supplementary file 1 — Figure S1. The optimal cutoff value was 39 mm/hour for ESR with OS according to X-tile. Abbreviations: ESR: erythrocyte sedimentation rate; OS: overall survival. (TIF 158 kb) [file 12885_2018_4914_MOESM1_ESM.tif]

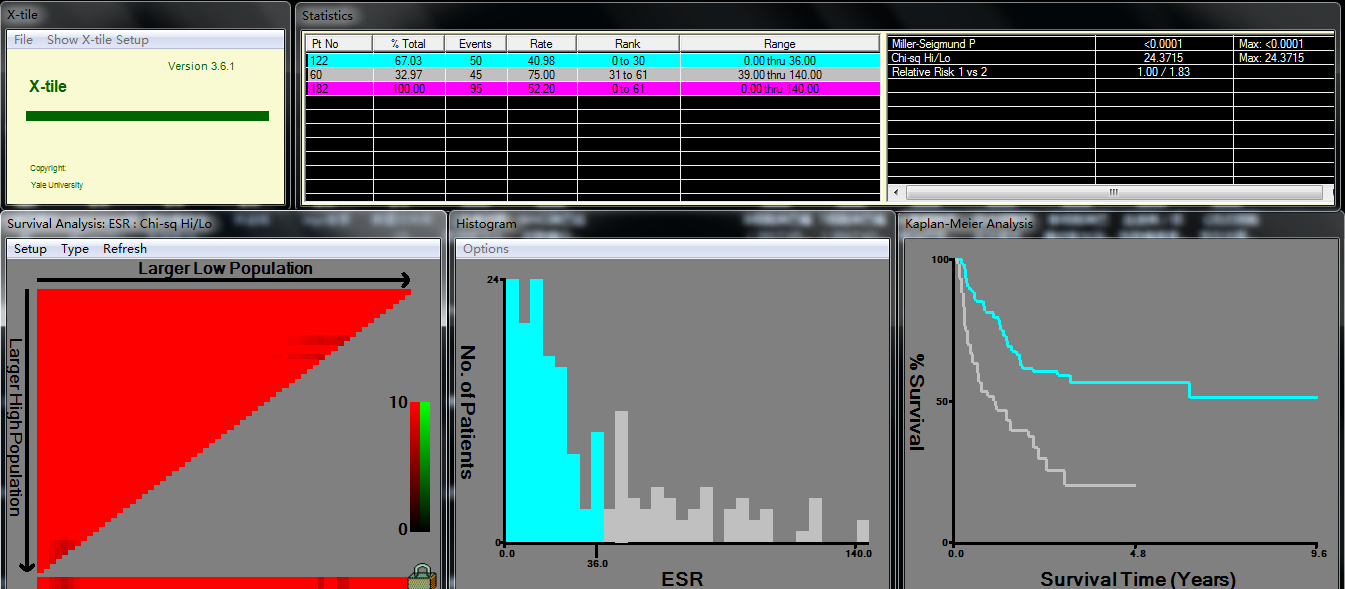

Supplement: Supplementary file 2 — Figure S2. The optimal cutoff value was 36 mm/hour for ESR with PFS according to X-tile. Abbreviations: ESR: erythrocyte sedimentation rate; PFS: progression-free survival. (TIF 154 kb) [file 12885_2018_4914_MOESM2_ESM.tif]
